# Supplementary material for: Diversity and Potential Cross-Species Transmission of Rotavirus A in Wild Animals in Yunnan, China
Source: Microorganisms. 2025 Jan 13;13(1):145. doi: 10.3390/microorganisms13010145 (PMC11767859; doi:10.3390/microorganisms13010145)
Supplement: Supplementary file 1 [file microorganisms-13-00145-s001.zip › Table S1.pdf]

Table S1. The primers and PCR product lengths for the RVA VP4 gene, VP7 gene, and host gene screening.

Gene		Name	Sequence (5' – 3')	Product Size (bp)
VP7/G	Round1	RV-A-F1	ATGCTCCTTTTRATGTATGGTA	333
		RV-A-R1	GTNGGCCATCCTTTNGT	
	Round2	RV-A-F2	ATGTATGGTATTGAATATACCAC	193
		RV-A-R2	GTRTCCATDGATCCAGTNATTGG	
VP4/P	Round1	RV-A4-F1	GGCTATAAAATGGYTTCNYT	
		RV-A4-R1	ARYADCCARTAATCRNYDRGTG	325
	Round2	RV-A4-F2	ATGGYTTCNYTMATTTATAGACA	
		RV-A4-R2	GNTGGYTGATAWGGACCRTCKA	175
Cytochrome b	Round1	cybF1-14724	CGAAGCTTGATATGAAAAACCATCGTT	
		cybR-15915	GGAATTCATCTCTCCGGTTTACAAGA	1191
	Round2	cybF2-15162	GCAAGCTTCTACCATGAGGACAAATATC	
		cybR-15915	GGAATTCATCTCTCCGGTTTACAAGA	753
