# Supplementary material for: Diversity and Potential Cross-Species Transmission of Rotavirus A in Wild Animals in Yunnan, China
Source: Microorganisms. 2025 Jan 13;13(1):145. doi: 10.3390/microorganisms13010145 (PMC11767859; doi:10.3390/microorganisms13010145)
Supplement: Supplementary file 1 [file microorganisms-13-00145-s001.zip › Table S2.pdf]

Table S2. The GenBank accession numbers of the strains

No.	Strains name + Gene	GenBank accession numbers
1	RVA/Rat/China/LH1221/2022/G3P[10] VP1	PQ724848
2	RVA/Rat/China/LH1221/2022/G3P[10] VP2	PQ724849
3	RVA/Rat/China/LH1221/2022/G3P[10] VP3	PQ724850
4	RVA/Rat/China/LH1221/2022/G3P[10] VP4	PQ724851
5	RVA/Rat/China/LH1221/2022/G3P[10] VP6	PQ724852
6	RVA/Rat/China/LH1221/2022/G3P[10] VP7	PQ724853
7	RVA/Rat/China/LH1221/2022/G3P[10] NSP1	PQ724854
8	RVA/Rat/China/LH1221/2022/G3P[10] NSP2	PQ724855
9	RVA/Rat/China/LH1221/2022/G3P[10] NSP3	PQ724856
10	RVA/Rat/China/LH1221/2022/G3P[10] NSP4	PQ724857
11	RVA/Porcine/China/ZT5159/2023/G5P[13] VP1	PQ724858
12	RVA/Porcine/China/ZT5159/2023/G5P[13] VP2	PQ724859
13	RVA/Porcine/China/ZT5159/2023/G5P[13] VP3	PQ724860
14	RVA/Porcine/China/ZT5159/2023/G5P[13] VP6	PQ724861
15	RVA/Porcine/China/ZT5159/2023/G5P[13] NSP1	PQ724862
16	RVA/Porcine/China/ZT5159/2023/G5P[13] NSP2	PQ724863
17	RVA/Porcine/China/ZT5159/2023/G5P[13] NSP3	PQ724864
18	RVA/Porcine/China/ZT5159/2023/G5P[13] NSP4	PQ724865
19	RVA/Porcine/China/ZT5159/2023/G5P[13] NSP5	PQ724866
20	RVA/Porcine/China/ZT2130/2023/G3P[13]VP1	PQ724867
21	RVA/Porcine/China/ZT2130/2023/G3P[13]VP6	PQ724868
22	RVA/Porcine/China/ZT2130/2023/G3P[13]NSP1	PQ724869
23	RVA/Porcine/China/ZT2130/2023/G3P[13]NSP2	PQ724870
24	RVA/Porcine/China/ZT2130/2023/G3P[13]NSP3	PQ724871
25	RVA/Porcine/China/ZT2130/2023/G3P[13]NSP4	PQ724872
26	RVA/Porcine/China/ZT2130/2023/G3P[13]NSP5	PQ724873
27	RVA/Bat/China/LC9/2024/G3P[3] VP4	PQ724874
28	RVA/Bat/China/LC10/2024/G3P[3] VP4	PQ724875
29	RVA/Bat/China/LC27/2024/G3P[3] VP4	PQ724876
30	RVA/Bat/China/LC28/2024/G3P[3] VP4	PQ724877
31	RVA/Bat/China/LC32/2024/G3P[3] VP4	PQ724878
32	RVA/Bat/China/LC53/2024/G3P[3] VP4	PQ724879
33	RVA/Bat/China/LC70/2024/G3P[3] VP4	PQ724880
34	RVA/Bat/China/LC89/2024/G3P[3] VP4	PQ724881
35	RVA/Bat/China/LC129/2024/G3P[3] VP4	PQ724882
36	RVA/Bat/China/LC130/2024/G3P[3] VP4	PQ724883
37	RVA/Bat/China/LC133/2024/G3P[3] VP4	PQ724884
38	RVA/Bat/China/LC1145/2024/G3P[3] VP4	PQ724885
39	RVA/Bat/China/NJ235/2024/G3P[3] VP4	PQ724886
40	RVA/Bat/China/NJ236/2024/G3P[3] VP4	PQ724887
41	RVA/Bat/China/LC9/2024/G3P[3] VP7	PQ724888
42	RVA/Bat/China/LC10/2024/G3P[3] VP7	PQ724889

43	RVA/Bat/China/LC27/2024/G3P[3] VP7	PQ724890
44	RVA/Bat/China/LC28/2024/G3P[3] VP7	PQ724891
45	RVA/Bat/China/LC32/2024/G3P[3] VP7	PQ724892
46	RVA/Bat/China/LC53/2024/G3P[3] VP7	PQ724893
47	RVA/Bat/China/LC70/2024/G3P[3] VP7	PQ724894
48	RVA/Bat/China/LC89/2024/G3P[3] VP7	PQ724895
49	RVA/Bat/China/LC129/2024/G3P[3] VP7	PQ724896
50	RVA/Bat/China/LC130/2024/G3P[3] VP7	PQ724897
51	RVA/Bat/China/LC133/2024/G3P[3] VP7	PQ724898
52	RVA/Bat/China/LC1145/2024/G3P[3] VP7	PQ724899
53	RVA/Bat/China/NJ235/2024/G3P[3] VP7	PQ724900
54	RVA/Bat/China/NJ236/2024/G3P[3] VP7	PQ724901
55	RVA/Avian/China/Dali8/2023/G34P[17]VP4	PQ724902
56	RVA/Avian/China/Dali9/2023/G34P[17]VP4	PQ724903
57	RVA/Avian/China/Dali8/2023/G34P[17]VP7	PQ724904
58	RVA/Avian/China/Dali9/2023/G34P[17]VP7	PQ724905
59	RVA/Porcine/China/ZT10/2023/G5P13VP4	PQ724906
60	RVA/Porcine/China/ZT11/2023/G5P13VP4	PQ724907
61	RVA/Porcine/China/ZT40/2023/G5P13VP4	PQ724908
62	RVA/Porcine/China/ZT41/2023/G5P13VP4	PQ724909
63	RVA/Porcine/China/ZT1120/2023/G5P13VP4	PQ724910
64	RVA/Porcine/China/ZT2130/2023/G5P13VP4	PQ724911
65	RVA/Porcine/China/ZT5159/2023/G5P13VP4	PQ724912
66	RVA/Porcine/China/ZT10/2023/G5P13VP7	PQ724913
67	RVA/Porcine/China/ZT11/2023/G5P13VP7	PQ724914
68	RVA/Porcine/China/ZT40/2023/G5P13VP7	PQ724915
69	RVA/Porcine/China/ZT41/2023/G5P13VP7	PQ724916
70	RVA/Porcine/China/ZT1120/2023/G5P13VP7	PQ724917
71	RVA/Porcine/China/ZT2130/2023/G5P13VP7	PQ724918
72	RVA/Porcine/China/ZT5159/2023/G5P13VP7	PQ724919
73	RVA/Porcine/China/ZT1120/2023/G5P[13] VP1	PQ732191
74	RVA/Porcine/China/ZT1120/2023/G5P[13] VP2	PQ732192
75	RVA/Porcine/China/ZT1120/2023/G5P[13] VP3	PQ732193
76	RVA/Porcine/China/ZT1120/2023/G5P[13] VP6	PQ732194
77	RVA/Porcine/China/ZT1120/2023/G5P[13] NSP1	PQ732195
78	RVA/Porcine/China/ZT1120/2023/G5P[13] NSP2	PQ732196
79	RVA/Porcine/China/ZT1120/2023/G5P[13] NSP3	PQ732197
80	RVA/Porcine/China/ZT1120/2023/G5P[13] NSP4	PQ732198
81	RVA/Porcine/China/ZT1120/2023/G5P[13] NSP5	PQ732199
