# Supplementary material for: Diversity and Potential Cross-Species Transmission of Rotavirus A in Wild Animals in Yunnan, China
Source: Microorganisms. 2025 Jan 13;13(1):145. doi: 10.3390/microorganisms13010145 (PMC11767859; doi:10.3390/microorganisms13010145)
Supplement: Supplementary file 1 [file microorganisms-13-00145-s001.zip › Table S3.pdf]

Table S3. Mammal samples tested for RVA in this study

**Animal species**<br>**Mammal**		
**Bats**	**No. of Collected Samples**	**No. of RVA Positive Samples（%+）**
Rhinolophus monoceros	2	0
Rhinolophus pusillus	5	0
Rhinolophus sinicus	12	0
Rhinolophus affinis	28	0
Rhinolophus thomasi	9	0
Rhinolophus pearsonii	1	0
Pipistrellus pipistrellus	10	0
Miniopterus pusillus	53	0
Hipposideros pomona	121	10（8.26%）
Hipposideros larvatus	22	3（13.64%）
Hipposideros armiger	29	0
Rousettus leschenaultii	6	0
Eonycteris spelaea	61	1（1.63%）
Rousettus amplexicaudatus	49	0
**Sub-total**	492	14（2.85%）
Wild boar		
Sus scrofa	90	7（7.78%）
**Sub-total**	90	7（7.78%）
**Rodent**		
Rattus tanezum	188	1（0.53%）
Rattus nitidus	12	0
Rattus norvegicus	54	0
**Sub-total**	254	1（0.39%）
**Others Mamml**		
Hystrix branchyura	29	0
Helarctos malayanus	53	0
Muntiacus muntjak	26	0
Ailurus fulgens	8	0
Nycticebus bengalensis	2	0
Macaca mulatta	13	0
Sciurus	15	0
Cervus nippon	18	0
Moschus berezovskii	23	0
Paguma larvata	5	0
Prionailurus bengalensis	12	0
Sorex araneus Linnaeus	18	0
**Sub-total**	222	0
