# Supplementary material for: Diversity and Potential Cross-Species Transmission of Rotavirus A in Wild Animals in Yunnan, China
Source: Microorganisms. 2025 Jan 13;13(1):145. doi: 10.3390/microorganisms13010145 (PMC11767859; doi:10.3390/microorganisms13010145)
Supplement: Supplementary file 1 [file microorganisms-13-00145-s001.zip › Table S4.pdf]

Table S4. Birds tested for RVA in this study

Animal species<br>Birds	No. of Collected Samples	No. of RVA Positive Samples（%+）
Columbiformes	13	0
Gruiformes	18	0
Galliformes	15	0
Cuculiformes	12	0
Passeriformes	143	2（1.39%）
Pelecaniformes	14	0
Ciconiiformes	133	0
Charadriiformes	189	0
Anseriformes	111	0
**Sub-total**	648	2（0.31%）
