# Supplementary material for: Diversity and Potential Cross-Species Transmission of Rotavirus A in Wild Animals in Yunnan, China
Source: Microorganisms. 2025 Jan 13;13(1):145. doi: 10.3390/microorganisms13010145 (PMC11767859; doi:10.3390/microorganisms13010145)
Supplement: Supplementary file 1 [file microorganisms-13-00145-s001.zip › Table S5.pdf]

Table S5. Transcription assembly library-related information

Library	Strains name	Animal species
pool 01	RVA/Pronice/CHN/2023/ZT11-20/G9P[13]	
pool 02	RVA/Pronice/CHN/2023/ZT21-30/G3P[13]	Sus scrofa
pool 03	RVA/Pronice/CHN/2023/ZT51-59/G5P[13]	
pool 04	RVA/Rat/CHN/2022/LH12-21/G3P[10]	Rattus tanezum
