# Supplementary material for: Diversity and Potential Cross-Species Transmission of Rotavirus A in Wild Animals in Yunnan, China
Source: Microorganisms. 2025 Jan 13;13(1):145. doi: 10.3390/microorganisms13010145 (PMC11767859; doi:10.3390/microorganisms13010145)
Supplement: Supplementary file 1 [file microorganisms-13-00145-s001.zip › Table S6.pdf]

Table S6 Nucleotide sequence similarity analysis of wild boar genomic segments

| Strain | Gene | Genotype | Strain exhibiting highest identity | | | | |
|---|---|---|---|---|---|---|---|
| | | | Strain name | Accession no. | Nucleotide identity(%) | Host | Country |
| ZT11-20 | VP7 | G9 | Rotavirus long E-type | AF501578 | 98.16 | Human | India: Manipur |
| | VP4 | P13 | RVA/Pig-wt/CHN/GD107/2023 | PP580379 | 95.02 | Swine | China |
| | VP6 | I5 | Rotavirus JSNJ2019 | PP100165 | 98.17 | Porcine | China |
| | VP1 | R2 | Bovine rotavirus HLJ-H3/2022/CHN | OQ807041 | 91.59 | Cow | China |
| | VP2 | C2 | Rotavirus sp. GS13 | PP997454 | 95.90 | Sheep | China |
| | VP3 | M1 | Giant panda rotavirus A CH-1 | HQ641295 | 93.34 | Ailuropoda melanoleuca | China |
| | NSP1 | A8 | Human rotavirus A R479 | GU189555 | 97.63 | Homo sapiens | China |
| | NSP2 | N2 | RVA/Human-wt/THA/SKT-27/2012/G6P[14] | LC055554 | 99.53 | Homo sapiens | Thailand: Sukhothai |
| | NSP3 | T1 | RVA/Pig-tc/JPN/I-TP2/2021/G9P[23] | LC776486 | 97.69 | Sus scrofa domesticus | Japan |
| | NSP4 | E1 | Porcine rotavirus A LLP48 | KJ126820 | 98.18 | Sus scrofa | China |
| | NSP5 | H1 | Porcine rotavirus HP140 G6P[13] | DQ003299 | 99.04 | Porcine | India |
| ZT21-30 | VP7 | G3 | Rotavirus A DB/LT/2210241 | OR948017 | 96.90 | Sus scrofa | China |
| | VP4 | P13 | CHN/SD/LP3/2022/G9P[13] | OQ799714 | 98.69 | Porcine | China |
| | VP6 | I5 | RVA/Pig-wt/CHN/HBP445/2021/G9P[13] | OR683315 | 97.53 | Pig | China |
| | VP1 | R1 | RVA/Hu/RUS/Moscow-1P/2015 | MT876637 | 91.41 | Homo sapiens | Russia |
| | VP2 | C-X | | | | | |
| | VP3 | M-X | | | | | |
| | NSP1 | A8 | RVA/Pig-tc/CHN/SWU-1C/2018/G9P[13] | MK410289 | 98.22 | Porcine | China |
| | NSP2 | N1 | Rotavirus A GD | OR911931 | 97.59 | Swine | China |
| | NSP3 | T1 | RVA/Pig/China/SC11/2017/G9P[23] | MH624170 | 68.66 | Pig-let | China |
| | NSP4 | E1 | RVA/Pig-wt/MOZ/MZ-MPT-193/2016/G9P13 | MT784860 | 98.48 | Sus scrofa domesticus | Mozambique |
| | NSP5 | H1 | Human rotavirus A GX54 | KF041439 | 98.86 | Homo sapiens | China |
| ZT51-59 | VP7 | G5 | Human rotavirus A LL3354G5P[6] | EF159575 | 96.62 | Human | China |
| | VP4 | P13 | Porcine rotavirus HP140 G6P[13] | DQ003291 | 94.99 | Porcine | India |
| | VP6 | I5 | Porcine rotavirus JSNJ2019 G1P[7] | PP100165 | 96.76 | Porcine | China |
| | VP1 | R1 | Porcine rotavirus JC3 | ON381976 | 96.68 | Porcine | Anjing Fu |
| | VP2 | C1 | Rotavirus A FX17 | OM362097 | 95.51 | Sus scrofa | China |
| | VP3 | M1 | RVA/Human-wt/VNM/NT0599/2008/G4P[6] | LC095937 | 98.53 | Homo sapiens | Viet Nam |
| | NSP1 | A1 | RVA/Pig-wt/CHN/SCLSHL-2-3/2017/G9P[23] | MH137274 | 95.58 | Pig | China |
| | NSP2 | N1 | RVA/Pig-wt/CHN/GDFZ/2023/G9P23 | PP566182 | 97.36 | Swine | China |
| | NSP3 | T1 | RVA/Human-wt/CHN/R1954/2013/G4P[6] | KF726074 | 98.74 | Homo sapiens | China |
| | NSP4 | E1 | RVA/Pig-wt/MOZ/MZ-MPT-193/2016/G9P[13] | MT784860 | 96.41 | Sus scrofa domesticus | Mozambique |
| | NSP5 | H1 | RVA/Pig-wt/MOZ/MZ-MPT-193/2016/G9P[13] | MH137270 | 96.41 | Pig | China |
